# Large-Scale Low-Cost NGS Library Preparation Using a Robust Tn5 Purification and Tagmentation Protocol

**DOI:** 10.1534/g3.117.300257

**Published:** 2017-11-08

**Authors:** Bianca P. Hennig, Lars Velten, Ines Racke, Chelsea Szu Tu, Matthias Thoms, Vladimir Rybin, Hüseyin Besir, Kim Remans, Lars M. Steinmetz

**Affiliations:** * Genome Biology Unit, European Molecular Biology Laboratory, Heidelberg 69117, Germany; † Protein Expression and Purification Core Facility, European Molecular Biology Laboratory, Heidelberg 69117, Germany; ‡ Biochemistry Center Heidelberg, Heidelberg University, 69120, Germany; § Department of Genetics, Stanford University School of Medicine, California 94305; ** Stanford Genome Technology Center, Stanford University School of Medicine, Palo Alto, California 94304

**Keywords:** Tn5, tagmentation, single cell

## Abstract

Efficient preparation of high-quality sequencing libraries that well represent the biological sample is a key step for using next-generation sequencing in research. Tn5 enables fast, robust, and highly efficient processing of limited input material while scaling to the parallel processing of hundreds of samples. Here, we present a robust Tn5 transposase purification strategy based on an N-terminal His_6_-Sumo3 tag. We demonstrate that libraries prepared with our in-house Tn5 are of the same quality as those processed with a commercially available kit (Nextera XT), while they dramatically reduce the cost of large-scale experiments. We introduce improved purification strategies for two versions of the Tn5 enzyme. The first version carries the previously reported point mutations E54K and L372P, and stably produces libraries of constant fragment size distribution, even if the Tn5-to-input molecule ratio varies. The second Tn5 construct carries an additional point mutation (R27S) in the DNA-binding domain. This construct allows for adjustment of the fragment size distribution based on enzyme concentration during tagmentation, a feature that opens new opportunities for use of Tn5 in customized experimental designs. We demonstrate the versatility of our Tn5 enzymes in different experimental settings, including a novel single-cell polyadenylation site mapping protocol as well as ultralow input DNA sequencing.

Next generation sequencing (NGS) methods have revolutionized biology by providing detailed insights into genome organization, the regulation of gene expression, and genetic variability. However, recent studies have revealed that studying the transcriptomes and epigenomes of entire cell populations can mask tissue complexity. In this respect, single-cell approaches have led to the discovery and characterization of multiple new cell types. In parallel, clinicians are calling for a paradigm shift toward personalized medicine based on information from each patient’s genome (Esplin *et al.* 2014; Giladi and Amit 2017; Ashley 2016). However, both developments necessitate the profiling of hundreds to thousands of samples (Velten *et al.* 2017; Buenrostro *et al.* 2015; Ramskold *et al.* 2012; Jaitin *et al.* 2014; Paul *et al.* 2015; Nagano *et al.* 2013; Grun *et al.* 2015).

The most rapid and scalable NGS library preparation strategy available to date is based on a hyperactive version of the Tn5 transposase (Illumina; Adey *et al.* 2010). The corresponding wild-type transposable element is present in bacteria and contains two nearly identical but inverted elements, IS50L and IS50R, with IS50R encoding the Tn5 transposase [reviewed in Reznikoff (2008)]. Tn5 catalyzes the translocation of transposable elements by a “cut-and-paste” mechanism that relies on a Tn5-homodimer binding to the outside ends (OE), which are two identical 19 bp inverted repeat sequences present at the outer sides of IS50 elements (Johnson and Reznikoff 1983; Sasakawa *et al.* 1983). Notably, the target integration locus is selected with only very modest genomic sequence motif requirements, *i.e.*, a mild tendency toward GC-rich regions (Shevchenko *et al.* 2002; Reznikoff 2008; Goryshin *et al.* 1998).

The efficiency of this process and the activity of the Tn5 transposase are regulated on many levels, resulting in a rather low transposition frequency *in vivo*. However, extensive studies over many years have resulted in the generation of a hyperactive version of Tn5 by introducing three point mutations—E54K, M56A, and L372P—where both the N-terminal M56A as well as the C-terminal L372P mutations reduce inhibitory effects on the activity of Tn5 (Goryshin and Reznikoff 1998; Weinreich *et al.* 1994). In parallel, the E54K mutation increases the DNA-binding efficiency of Tn5 to the OE (Zhou and Reznikoff 1997). Furthermore, Zhou and colleagues have reported increased binding efficiency of the E54K mutant to a mosaic sequence of the natural transposon OE and inside end elements, the mosaic end (ME) element, *in vitro* (Zhou *et al.* 1998).

Its relatively simple mode of action raised interest in the scientific community in using Tn5 as a molecular tool for NGS library preparation. Illumina developed the tagmentation protocol, in which a modified Tn5 enzyme cuts double-stranded DNA and concurrently ligates the linker sequences that are required for Illumina sequencing to both ends. Due to its high efficiency, the low input concentration needed, and its limited hands-on time, this tagmentation protocol not only meets the requirements of NGS library construction, but also for the processing of thousands of samples in parallel (Adey *et al.* 2010).

Recently, the Sandberg laboratory published a protocol describing the purification of hyperactive Tn5 and subsequent tagmentation-based library preparation (Picelli *et al.* 2014a). In this study, the authors used a Tn5 construct containing the E54K and L372P mutations previously described by Martínez-García *et al.* (2011). They loaded the enzyme with a synthetic oligonucleotide containing the double-stranded 19-bp ME sequence and a single-stranded 5′ overhang. Tn5 bound to the 19 bp ME region of the oligonucleotide and ligated it to the input DNA on both sides of the cut site. The single-stranded 5′ ends served as templates for index adapter primers during PCR-based NGS library preparation. When using this protocol, we encountered difficulties in purifying the Tn5-intein-CBD fusion construct and failed to reproducibly obtain libraries for NGS. We therefore attempted to improve the Tn5 purification procedure.

Here, we report a revised and improved Tn5 purification protocol that is reproducible across institutes. Our robust tagmentation protocol uses solely homemade or inexpensive commercially available reagents and reduces the costs of tagmentation-based library generation by up to 70-fold compared to the commercially available kit. We have validated the quality of the NGS libraries processed with our in-house Tn5 enzymes and demonstrated their suitability for various experimental designs, including single-cell full-length RNA-seq, Drop-Seq (Macosko *et al.* 2015), and ultralow input sequencing. We also report a novel single-cell polyadenylation site mapping protocol based on custom tagmentation linkers. By comparing two Tn5 molecules that reproducibly generate high-quality NGS libraries, but vary in their characteristic features, we pave the way toward further development of novel applications for Tn5.

## Materials and Methods

Detailed protocols are provided in the Supplemental Material, Supplemental Methods in File S1. All oligonucleotides used in this study were ordered at HPLC grade from Sigma Aldrich, Germany. Index oligonucleotide sequences were adapted from Illumina: Oligonucleotide sequences © 2007–2013 Illumina, Inc. All rights reserved.

### Subcloning of the hyperactive Tn5 allele into pETM11-Sumo3

The pTXB1 plasmid carrying the Tn5-intein-CBD fusion construct with the hyperactive Tn5 allele containing the E54K and L372P mutations was a generous gift from the Rickard Sandberg lab. This plasmid was used as template to PCR-amplify the hyperactive Tn5 allele. We replaced the catalytic cysteine of the GyrA intein protein after the terminal Tn5 isoleucine, introduced by Picelli *et al.* (2014a), with the stop codon TAA. The PCR product was *Bam*HI- and *Hin*dIII-digested and cloned into the pETM11-Sumo3 vector (European Molecular Biology Laboratory [EMBL], available upon request). This cloning strategy introduced an additional serine residue between the Sumo3 protein and the Tn5 construct, and resulted in a His_6_-Sumo3-Tn5 fusion protein. The coding sequence of the Tn5 transposase was validated via Sanger sequencing.

### Expression and purification of homemade Tn5

The pETM11-Sumo3-Tn5 plasmid was transformed into *Escherichia coli* BL21(DE3) codon + RIL cells (Stratagene). Cells were grown in LB supplemented with kanamycin and chloramphenicol at 37° until OD_600_ ∼0.5. The temperature was then lowered to 18° and expression of Tn5 was induced by the addition of 0.2 mM IPTG. The cells were then grown overnight at 18° and harvested by centrifugation. The cell pellet was resuspended in running buffer (20 mM HEPES-NaOH pH 7.2, 800 mM NaCl, 20 mM imidazole, 1 mM EDTA, 2 mM DTT, and 10% glycerol) supplemented with cOmplete protease inhibitors (Roche) and lysed via sonication. Polyethyleneimine (PEI) pH 7.2 was added dropwise to a final concentration of 1% to remove nucleic acids. The cleared lysate was loaded onto a cOmplete His-tag purification column (Roche) and the His_6_-Sumo3-Tn5 was eluted in running buffer containing 300 mM imidazole. To remove the fusion tag, His_6_-tagged SenP2 protease was added to the elution fractions. The sample was digested overnight at 4° while being dialyzed back to running buffer. The next morning, the dialyzed sample was loaded again onto a cOmplete His-tag purification column (Roche) and the untagged Tn5 was collected in the flow through. The concentrated flow through was then loaded onto a Superdex200 Increase 10/300 GL column (GE Healthcare) equilibrated with 50 mM Tris pH 7.5, 800 mM NaCl, 0.2 mM EDTA, 2 mM DTT, and 10% glycerol. Elution fractions corresponding to the Tn5 dimer peak were pooled and aliquoted. The identity of both Tn5 variants was verified by mass spectrometry (Figure S1 and Supplemental Methods in File S1). Final Tn5 samples were stored at −20° in 25 mM Tris pH 7.5, 800 mM NaCl, 0.1 mM EDTA, 1 mM DTT, and 50% glycerol.

### Isothermal titration calorimetry (ITC) experiments

Binding of linker DNA to Tn5_E54K,L372P_ and Tn5_R27S,E54K,L372P_ was studied in ITC buffer (50 mM Tris pH 7.5, 800 mM NaCl, 0.1 mM EDTA, and 5% glycerol) using a MicroCal ITC_200_ System (MicroCal). All proteins and DNA samples were dialyzed overnight against ITC buffer at 4°. Titration was performed at 25° by stepwise addition of 100 µM DNA solution to the 4 µM protein solution in the cell. ITC data were corrected for dilution heat and analyzed using the MicroCal Origin software package.

### Loading of in-house-produced Tn5

Tn5 was loaded with the previously reported linker oligonucleotides Tn5ME-A/Tn5MErev and Tn5ME-B/Tn5MErev (Picelli *et al.* 2014a) by mixing equal amounts of each linker with Tn5 and incubating at 23° for 30–60 min. The loaded Tn5 was ready to use without removal of unbound linkers and dilutions were performed in nuclease-free water. Alternatively, loaded Tn5 can be supplemented with glycerol at a final concentration of 50% and stored at −20° for several days.

### Tagmentation-based library preparation for duplex index full-length RNA sequencing using in-house-produced Tn5

Tagmentation was performed in 10 mM Tris-HCl pH 7.5 (Sigma Aldrich), 10 mM MgCl_2_ (Sigma Aldrich), and 25% dimethylformamide (DMF) (Sigma Aldrich) using 100–200 pg cDNA. Excellent fragmentation of cDNA was achieved using Tn5 at a concentration of 20–40 ng/μl. Reactions were incubated at 55° for 3 min in a preheated thermocycler and either stopped with 0.2% SDS or heat inactivation (see Figure S6A and Supplemental Methods in File S1).

PCR amplification was performed using the KAPA HiFi HotStart polymerase or KAPA HiFi HotStart ReadyMix (see Figure S6B in File S1). Residual dNTPs, oligonucleotides, and polymerase were removed by subsequent bead purification using 1 vol of AMPureXP beads (Beckmann) following the manufacturer’s instructions. Final libraries were eluted with 10 μl nuclease-free water.

Tagmentation of genomic DNA was performed as described above. Note that the number of PCR cycles was increased when processing input material ≤0.5 pg/μl.

### Single-cell polyadenylation site mapping

#### cDNA preparation:

In this study, we performed all tagmentation experiments with cDNA obtained from extracted HeLa RNA processed with the previously described Smart-seq2 protocol (Picelli *et al.* 2013, 2014b) with the following modifications: we modified the oligo dT primer by introducing a four-nucleotide end identifier and an eight-nucleotide specific cell barcode. The end identifier was introduced only in the oligo dT but not in the template-switching primer (Picelli *et al.* 2014b), and thus allowed for specific amplification of the 3′ end of the cDNA without interfering with the common primer sequence present in both the oligo dT and template switching primer, which are needed for amplification of the cDNA library.

cDNA was processed from 15 pg extracted HeLa RNA, mimicking the RNA amount of a single cell. The amplified cDNA was purified using 1 vol of AMPureXP beads following the manufacturer’s instructions, then eluted in 15 μl nuclease-free water and stored at −20°. cDNA was quantified using the Qubit HS dsDNA (Invitrogen) and the size distribution and quality of the cDNA were analyzed with an Agilent High Sensitivity DNA kit and an Agilent 2100 Bioanalyzer following the manufacturer’s instructions.

#### Tagmentation:

To obtain 3′ RNA-specific libraries, we essentially used the above-described Tn5 loading and tagmentation protocol with the following changes: undiluted Tn5 stock was loaded exclusively with the Tn5ME-B/Tn5MErev linker. Subsequent PCR enrichment was performed using KAPA HiFi HotStart polymerase (Kapa Biosystems) supplemented with TMAC, i7 adapter index primer, and a custom PE1.Smart-seq2 primer. Importantly, we skipped the gap-filling step in the PCR program to prevent amplification of tagmented cDNA from the gene-coding region.

### Tagmentation using the Nextera XT DNA library preparation kit

Tagmentation using the Nextera XT DNA library preparation kit (Illumina) was performed following the manufacturer’s instructions. Purification of samples was performed using an identical volume of AMPureXP beads, according to the manufacturer’s instructions.

### Multiplexing and NGS of tagmented samples

Samples were multiplexed for sequencing by mixing equal concentrations of each library. The resulting volume was determined and an identical volume of AMPureXP beads was added. Purification was performed in accordance with the manufacturer’s instructions and the multiplexed library was eluted in 50 μl nuclease-free water. For size selection, 0.6 vol of AMPureXP were added to the sample and incubated at room temperature for 5 min, followed by separation of large fragments (beads) and small fragments (supernatant) using a magnet. The supernatant was transferred into a new tube (beads were discarded), and mixed with 0.2 vol of AMPureXP (with respect to the supernatant). Purification of the multiplexed library was performed according to the manufacturer’s instructions. The final library was eluted in 12 μl nuclease-free water. Samples were quantified using Qubit HS dsDNA. Quality control was performed using an Agilent 2100 Bioanalyzer and High Sensitivity DNA kit.

All libraries were sequenced on NextSeq platforms. Full-length RNA libraries were sequenced using the 75 bp paired end option with 75 cycles each from both the P5 and P7 sites using Illumina sequencing primers. Libraries were sequenced with either 75 cycles each from each end of the fragment (resulting in sequencing reads of 75 nt) or as an asymmetric run with all cycles from P7 (resulting in sequencing reads of 150 nt). Using the double indexing strategy, we obtained information about both i5 and i7 Illumina indices (each index has a length of 8 nt).

To map polyadenylation sites in single cells, 3′ RNA libraries were sequenced using the 150 bp paired end option as an asymmetric run. Read 1 with 14 cycles resulted in 14 nt long sequencing reads providing the cell barcode (6 nt) and UMI (8 nt, information not used), and was performed from the P5 site using a custom sequencing primer [poly(A)-seq primer, see File S1]. Read 2 was performed from the P7 site using the Illumina P7 sequencing primer with the remaining ∼290 cycles to identify the gene, resulting in sequencing reads of ∼290 nt. Read 2 was supposed to reach the polyadenylation site but was excluded from the analysis if the polyadenylation site was missing. A dedicated index read provided the i7 barcode (8 nt) and was used for demultiplexing into sample batches.

At least two technical replicates were sequenced per condition.

### Data analysis

Several batches of cDNA obtained from independent Smart-seq2 replicates were used in this study. All comparisons between Tn5 variants, Tn5 batches, and treatments were done on data obtained from an identical cDNA batch for each figure, unless stated differently.

For libraries created with the double indexing strategy, raw sequencing data were processed as described (Velten *et al.* 2017). In short, reads were demultiplexed using index read information and sequencing reads extending into the poly(A) tail were trimmed. Subsequently, all reads were aligned to the hg19 version of the human genome using GSNAP (Wu and Nacu 2010), and reads falling on annotated exons were counted using HTSeq (Anders *et al.* 2015). For polyadenylation site mapping libraries, data were processed as described (Velten *et al.* 2017), with small modifications. In short, samples were demultiplexed using the i7 index read and the first six bases of the first read. The first read was then discarded and all further processing was done on the second read. Reads extending into the poly(A) tail were trimmed and aligned to the hg19 version of the human genome using GSNAP, and alignments were filtered as previously described. We then created a table of read counts per genomic coordinate using a custom script based on HTSeq and call polyadenylation sites, as previously described.

All correlation coefficients shown are Pearson’s product–moment correlation coefficients. Clustering was performed using a correlation-based distance metric and complete linkage. The number of genes detected in NGS libraries was based on a minimum of five reads per barcode.

All scripts used for data analysis are available on GitHub (https://git.embl.de/velten/misc-pub/blob/master/HennigEtAl.R).

### Data availability

The pETM11-Sumo3-Tn5 plasmids carrying either the Tn5_E54K,L372P_ or Tn5_R27S,E54K,L372P_ allele are available upon request. The plasmid expressing the His_6_-tagged SenP2 protease is available upon request.

The data discussed in this manuscript have been deposited in NCBI’s Gene Expression Omnibus (GEO) and are accessible through GEO Series accession number GSE101520 (https://www.ncbi.nlm.nih.gov/geo/query/acc.cgi?acc=GSE101520).

File S1 includes Figures S1–S6, Table S1 which lists all primers used in this study, detailed protocols for mass spectrometry measurements, expression and purification of the Tn5 enzymes, as well as loading of the Tn5, tagmentation reaction, and subsequent PCR enrichment.

## Results

### Expression and purification of Tn5 transposase

**Figure 1:**
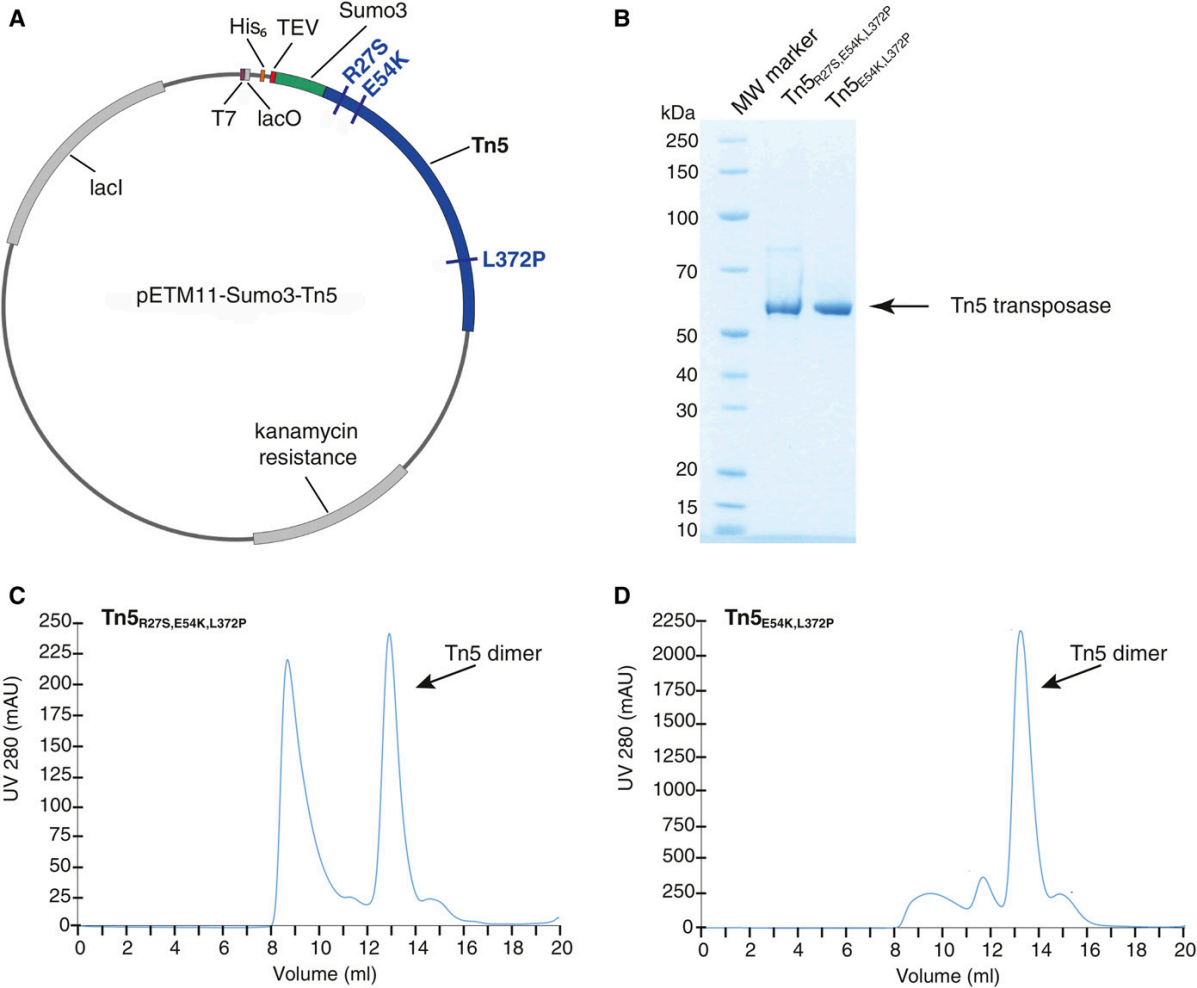
Purification of the His_6_-Sumo3-Tn5 construct. (A) Plasmid map of pETM11-Sumo3-Tn5 encoding the His_6_-Sumo3-Tn5 fusion protein. The locations of the point mutations in the Tn5 coding region (R27S, E54K, and L372P) are indicated. (B) SDS-PAGE analysis of the two His_6_-Sumo3 purified Tn5 proteins after size exclusion chromatography. The single band runs at a molecular weight of ∼53 kDa, corresponding to the Tn5 monomer. (C) Size exclusion chromatography (Superdex200 Increase 10/300 GL) profile of the purified Tn5_R27S,E54K,L372P_ transposase. The Tn5 dimer peak elutes at 12.9 ml, while the Tn5 aggregate elutes at 8.7 ml. (D) Size exclusion chromatography (Superdex200 Increase 10/300 GL) profile of the purified Tn5_E54K,L372P_ transposase. The Tn5 dimer peak is the main peak and elutes at 13.2 ml. MW, molecular weight; SDS-PAGE, sodium dodecyl sulfate-polyacrylamide gel electrophoresis.

In a previous study, Tn5 transposase was expressed from the pTXB1-Tn5 plasmid and purified via a C-terminal intein-CBP tag (Picelli *et al.* 2014a). We aimed to produce the Tn5 in-house following this protocol; however, this purification procedure proved to be intricate and we only obtained very low yields of the enzyme. Furthermore, the enzyme showed low activity and we failed to reproducibly obtain libraries when processing cDNA with this transposase. Therefore, we developed a new Tn5 purification strategy using the N-terminal His_6_-Sumo3 tag, which is a more common and straight forward protein purification method than using the intein tag. This is a very common and straight forward protein purification method that is likely to work robustly in most biochemistry laboratories. The hyperactive Tn5 allele carrying the E54K and L372P mutations, further referred to as Tn5_E54K,L372P_, was PCR-amplified from the pTXB1-Tn5 plasmid (Picelli *et al.* 2014a) and subcloned into the pETM11-Sumo3 vector (EMBL), resulting in the His_6_-Sumo3-Tn5 fusion construct (Figure 1A). The Tn5 sequence was validated via Sanger sequencing. One of the clones carried an additional point mutation in the DNA-binding domain that resulted in an amino acid substitution from arginine to serine (R27S), referred to as Tn5_R27S,E54K,L372P_ (Figure 1A). It is likely that this substitution was introduced during PCR amplification. The arginine is located in the DNA-binding domain of the Tn5 transposase and is involved in the binding of the linker oligonucleotide (Davies *et al.* 2000). Since the arginine to serine mutation leads to a loss of the positive charge, we hypothesized that the mutation might have an influence on the enzyme’s activity. In the following sections, we will compare the performance of both Tn5 enzymes during tagmentation-based library preparation.

The His_6_-Sumo3-Tn5 fusion protein was expressed in *E. coli* BL21(DE3) codon + RIL cells. *E. coli* DNA bound to the transposase was removed via PEI precipitation. After His-tag purification and cleaving of the His_6_-Sumo3 fusion tag, the untagged Tn5 was loaded on a size exclusion chromatography column to obtain a pure Tn5 dimer (Tn5 monomer ∼53 kDa, Figure 1, B–D) and stored at −20°.

### Initial activity tests with the homemade Tn5 enzymes

**Figure 2:**
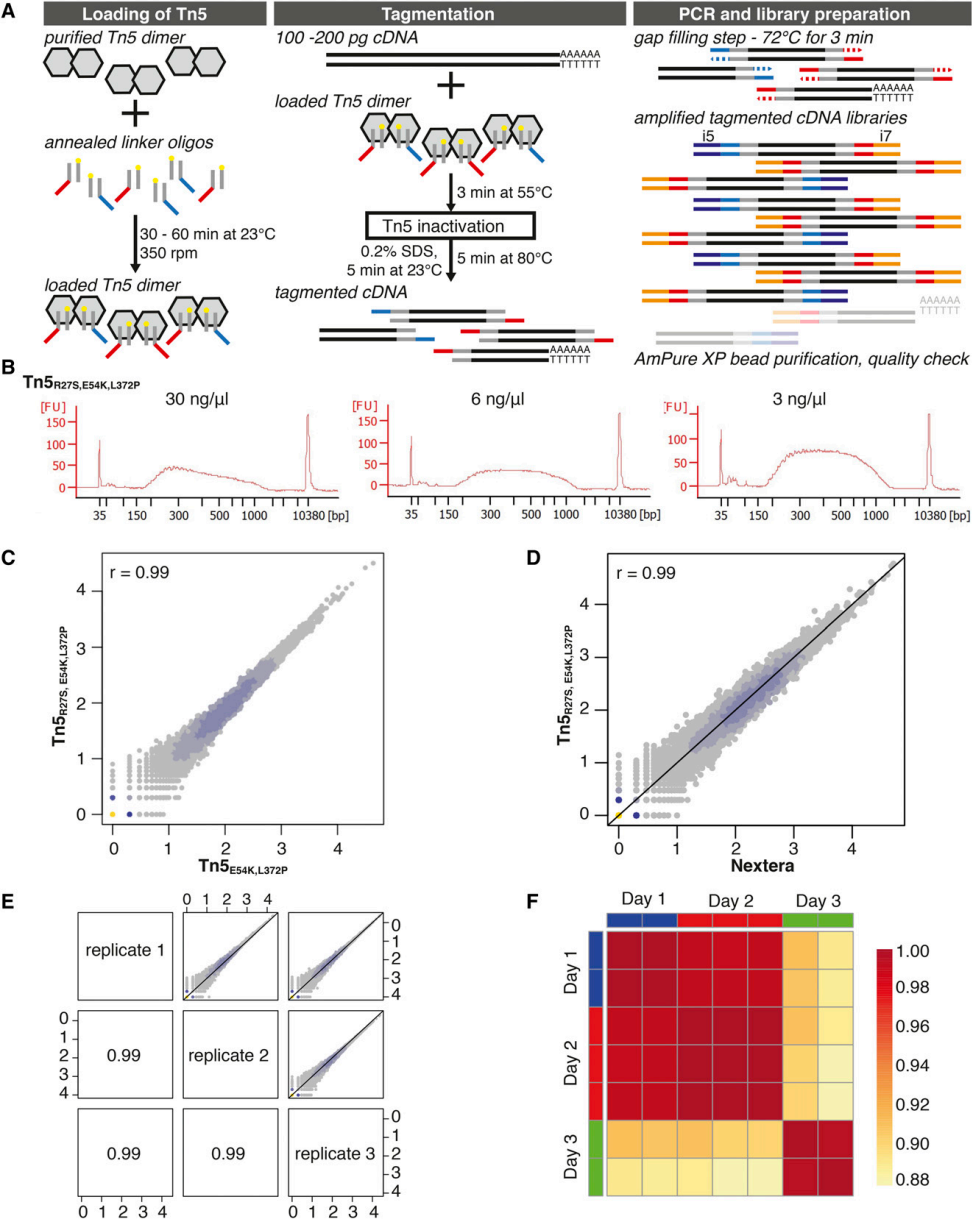
NGS-library preparation using the homemade Tn5 constructs. (A) Workflow of Tn5 loading, cDNA tagmentation, and subsequent NGS library preparation for duplex index (Illumina i5/i7) full-length cDNA sequencing. Tn5 molecules are shown as gray hexamers. The double-stranded part of the linker oligonucleotide, the mosaic element, is shown in gray with a yellow circle depicting the phosphorylated 3′ end. The 5′ overhangs as templates for the i5 or i7 index adapter primers are shown in red or blue, respectively. cDNA is shown as two parallel black lines with a 3′ poly(A) tail. The synthesis of the 5′ overhang complementary strand (gap-filling step during PCR amplification) is depicted as a dotted arrow. i5 index adapter primer is shown in dark blue, while i7 index adapter primer is shown in orange. Fragments that are lost during library preparation are transparent. (B) Bioanalyzer traces of NGS libraries processed with different concentrations of the in-house-produced Tn5_R27S,E54K,L372P_ using only homemade or inexpensive commercially available reagents for tagmentation and subsequent PCR reaction. Following the tagmentation protocol presented here, fragmentation of the cDNA works best when using Tn5_R27S,E54K,L372P_ at a concentration of 30 ng/μl. (C) Heat scatter plot showing the correlation of read counts between libraries processed with either in-house-produced Tn5_E54K,L372P_ or Tn5_R27S,E54K,L372P_. Data of three technical replicates per condition were pooled for this analysis. (D) Heat scatter plot showing the correlation of gene counts between libraries processed using either in-house-produced Tn5_R27S,E54K,L372P_ and the protocol presented here or the Nextera XT DNA library preparation kit following the manufacturer’s instructions. Data of three technical replicates per condition were pooled for this analysis. (E) Pairwise correlation of read counts between three technical replicates (samples processed from the same cDNA on the same day) when using homemade Tn5_R27S,E54K,L372P_ and the tagmentation protocol presented here. The Pearson correlation of *r* = 0.99 between all samples demonstrates high reproducibility of both the enzyme and the protocol. (F) Heat map analysis of gene counts in technical replicates processed from the same cDNA but on different days. The color code indicates the Pearson correlation between samples (see legend on the right side). NGS, next-generation sequencing; PCR, polymerase chain reaction; SDS, sodium dodecyl sulfate.

Figure 2A illustrates the tagmentation-based library preparation workflow using purified Tn5 constructs. Prior to tagmentation, the Tn5 enzyme is loaded with linker oligonucleotides containing the double-stranded 19 bp ME sequence and a single-stranded 5′ overhang (Picelli *et al.* 2014a). In a first attempt to test the activity of Tn5_E54K,L372P_ and Tn5_R27S,E54K,L372P_, we performed tagmentation reactions with reagents supplied in the Illumina Nextera XT DNA library preparation kit following the manufacturer’s instructions, but substituting the Nextera ATM enzyme with one of the two loaded in-house-produced Tn5 versions. As we did not know the activity range of these transposases, we performed several tagmentation reactions simultaneously with different dilutions of Tn5 ranging from 3 to 30 ng/μl, while keeping the cDNA concentrations constant (150–200 pg per reaction). Using Tn5_E54K,L372P_, we obtained libraries with an average fragment size of roughly 340 bp with all Tn5 concentrations used (Figure S2A in File S1). Fragment size distribution was similar when tagmenting with 30 ng/μl Tn5_R27S,E54K,L372P_, while higher dilutions of the enzyme with constant input cDNA concentrations resulted in larger fragment sizes (Figure S2B in File S1).

Overall, this initial experiment demonstrated that both purified Tn5 constructs are active and produce libraries suitable for sequencing from small amounts of input material. Interestingly, the hyperactive Tn5 version carrying the additional R27S mutation showed higher sensitivity toward the ratio of Tn5-to-cDNA molecules than the regular hyperactive Tn5_E54K,L372P_ enzyme.

### A robust tagmentation-based NGS library preparation protocol

In order to develop a tagmentation protocol that is completely independent of the Nextera XT DNA library preparation kit, we substituted all Nextera reagents with homemade or inexpensive commercial reagents in a stepwise manner. We found that a Tris-based tagmentation buffer at a final concentration of 10 mM Tris-HCl, 10 mM MgCl_2_, and 25% DMF resulted in robust and reproducible tagmentation of input cDNA. We used SDS inactivation and KAPA HiFi HotStart DNA polymerase (Kapa Biosystems) for subsequent PCR enrichment of the tagmented input cDNA to generate NGS libraries.

**Figure 3:**
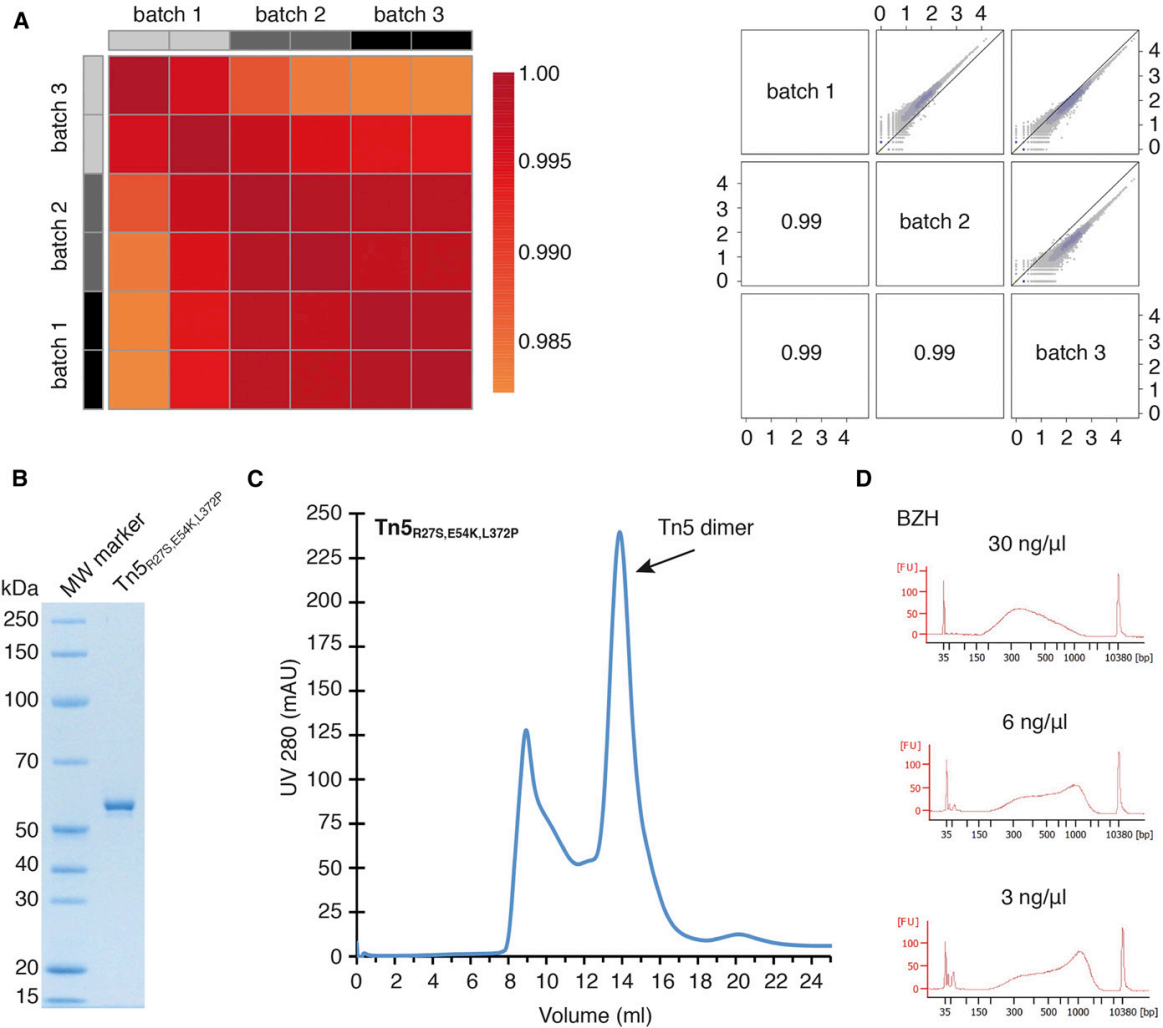
NGS library preparation using different batches of in-house-produced Tn5 and reproducibility of the Tn5 purification protocol across institutes. (A) Left panel: comparison of two technical replicates for each of the three Tn5 batches. The color code indicates the Pearson correlation between samples (see legend on the right side). Right panel: pairwise correlation of read counts between NGS-libraries processed with three different Tn5 batches. Two technical replicates were pooled for each condition. (B) SDS-PAGE analysis of His_6_-Sumo3 Tn5 enzyme after size exclusion chromatography, purified at BZH. The single band runs at a molecular weight of ∼53 kDa, corresponding to the Tn5 monomer. (C) Size exclusion chromatography (Superdex200 10/300 GL) profile of Tn5_R27S,E54K,L372P_ transposase purified at BZH. The Tn5 dimer peak elutes at 13.88 ml, while the Tn5 aggregate elutes at 8.93 ml. (D) Bioanalyzer traces of NGS-libraries processed with different concentrations of Tn5_R27S,E54K,L372P_ produced at BZH (see Figure 2B for libraries processed with Tn5 produced at EMBL). BZH, Heidelberg University Biochemistry Center; EMBL, European Molecular Biology Laboratory; MW, molecular weight; NGS, next-generation sequencing; SDS-PAGE, sodium dodecyl sulfate-polyacrylamide gel electrophoresis.

Using these conditions, we observed stable fragment size distribution of cDNA with a wide range of tested Tn5_E54K,L372P_ concentrations (3–30 ng/μl, Figure S3A in File S1). For Tn5_R27S,E54K,L372P_, the ideal library fragment size distribution was achieved using the enzyme at a concentration of 30 ng/μl, while higher dilutions of the enzyme increased the average fragment size (Figure 2B). These observations were in agreement with the results of the initial experiment described above (Figure S2B in File S1). The bigger fragments obtained when using the Nextera XT DNA library preparation buffer instead of the homemade Tris-based buffer for tagmentation with the Tn5_R27S,E54K,L372P_ enzyme are likely to result from faint differences in the ratio between cDNA input and enzyme (compare also Figure 3D). Importantly, libraries prepared with either of the transposases were of high quality and showed excellent Pearson correlations of *r* = 0.99 when processed from the same input cDNA (Figure 2C). We also compared the performance of our in-house Tn5 transposases with the Nextera XT DNA library preparation kit and found that NGS libraries using the same input cDNA processed with either the Tn5_R27S,E54K,L372P_ or the commercially available kit correlated very well with a Pearson’s correlation coefficient of *r* = 0.99 (Figure 2D and Figure S3B in File S1).

Using the Tn5_R27S,E54K,L372P_-processed libraries as an example, more detailed evaluation of the sequencing data demonstrated good raw read quality with a high percentage of reads uniquely aligning to the human genome (median across all experiments 87% of reads, first quartile: 86% and third quartile: 88%). On average, 9594 genes were detected per sample (median: 9740, first quartile: 9470, and third quartile: 9760), which is remarkable considering the limited amount of input material of ∼150–200 pg amplified cDNA processed from as little as 15 pg HeLa RNA [*e.g.*, see Hicks *et al.* (2015)]. We further validated the reproducibility of the gene expression analysis for both Tn5 variants and the Nextera XT DNA library preparation kit (Figure S3C in File S1). We obtained good coverage over the entire gene-coding region (Figure S3D in File S1) consistent with the full-length cDNA library preparation protocol. Importantly, we obtained excellent correlations between samples processed in the same experiment with a Pearson’s correlation coefficient of *r* = 0.99 (Figure 2E). We detected slightly lower correlation of detected genes when comparing samples prepared on different days, known as batch effects. However, the correlation coefficients between samples always remained above 0.88, and typically above 0.96 (Figure 2F), demonstrating high reproducibility between libraries processed on different days.

Taken together, our data demonstrate that we have developed a robust protocol for tagmentation of low cDNA amounts (*e.g.*, from single cells) and that both of our homemade Tn5 transposases work equally well for NGS library preparation. As library fragment size distribution only correlated with the tested Tn5-to-input ratios for Tn5_R27S,E54K,L372P_, we performed all following experiments with this enzyme and used fragment size distribution as an indicator for the activity of the enzyme, thereby inferring the quality and reproducibility of Tn5 purification and tagmentation reactions.

### Reproducibility of the His_6_-Sumo3-Tn5 purification

In the course of this study we produced three independent Tn5_R27S,E54K,L372P_ batches. Stock aliquots were stored for up to 14 months at −20° without any loss of activity or library quality (*r* = 0.99, Figure S3E in File S1). To compare the activity of the different Tn5_R27S,E54K,L372P_ purifications, we performed tagmentation experiments using the same input cDNA with a range of Tn5_R27S,E54K,L372P_ dilutions from all three batches. All batches showed comparable activity, as seen in the typical fragment size distribution patterns when using different concentrations of the Tn5 enzyme (3–30 ng/μl). Sequencing of the resulting libraries revealed pairwise Pearson correlations of *r* > 0.985 between samples processed with different Tn5 batches (Figure 3A), demonstrating that all three batches of in-house-produced Tn5 were of equal quality.

As we aimed to develop a Tn5 purification protocol that can be used across institutes, we passed our pETM11-Sumo3-Tn5_R27S,E54K,L372P_ plasmid on to a group at the Heidelberg University Biochemistry Center (BZH), who then independently repeated our expression and purification protocol. Importantly, following our protocol, the purification of the Tn5_R27S,E54K,L372P_ construct worked well in their hands (Figure 3, B and C). The activity and functionality of the Tn5 protein generated at BZH was then compared to the Tn5 protein purified at EMBL. We performed tagmentation on the same input cDNA using the EMBL- or BZH-purified Tn5 enzymes at a wide range of concentrations. Bioanalyzer profiles revealed comparable fragment size distribution of the NGS libraries at Tn5 dilutions ranging from 3 to 30 ng/μl (Figure 3D), demonstrating high reproducibility of the expression and purification of the His_6_-Sumo3-Tn5 construct across institutes.

### 3′ RNA Seq protocol using the Tn5_R27S,E54K,L372P_ enzyme

Thus far, we have demonstrated that our homemade Tn5 enzymes can be used to generate high-quality NGS libraries, and that our purification strategy using the His_6_-Sumo3 fusion tag is robust and reproducible across institutes. An important advantage of our in-house Tn5 is the possibility to load the enzyme with any desired linker oligonucleotide, allowing for customized experimental design. We leveraged this feature to develop a method suitable for the mapping of polyadenylation sites in single cells. Recent single-cell studies have revealed that cellular heterogeneity does not only involve varying levels of gene expression but also differential choice of polyadenylation sites in different cells, resulting in 3′ isoform heterogeneity between cells (Velten *et al.* 2015). Such approaches provide new insights into biological diversity at the cellular level; however, the underlying protocols are often time-consuming and costly.

**Figure 4:**
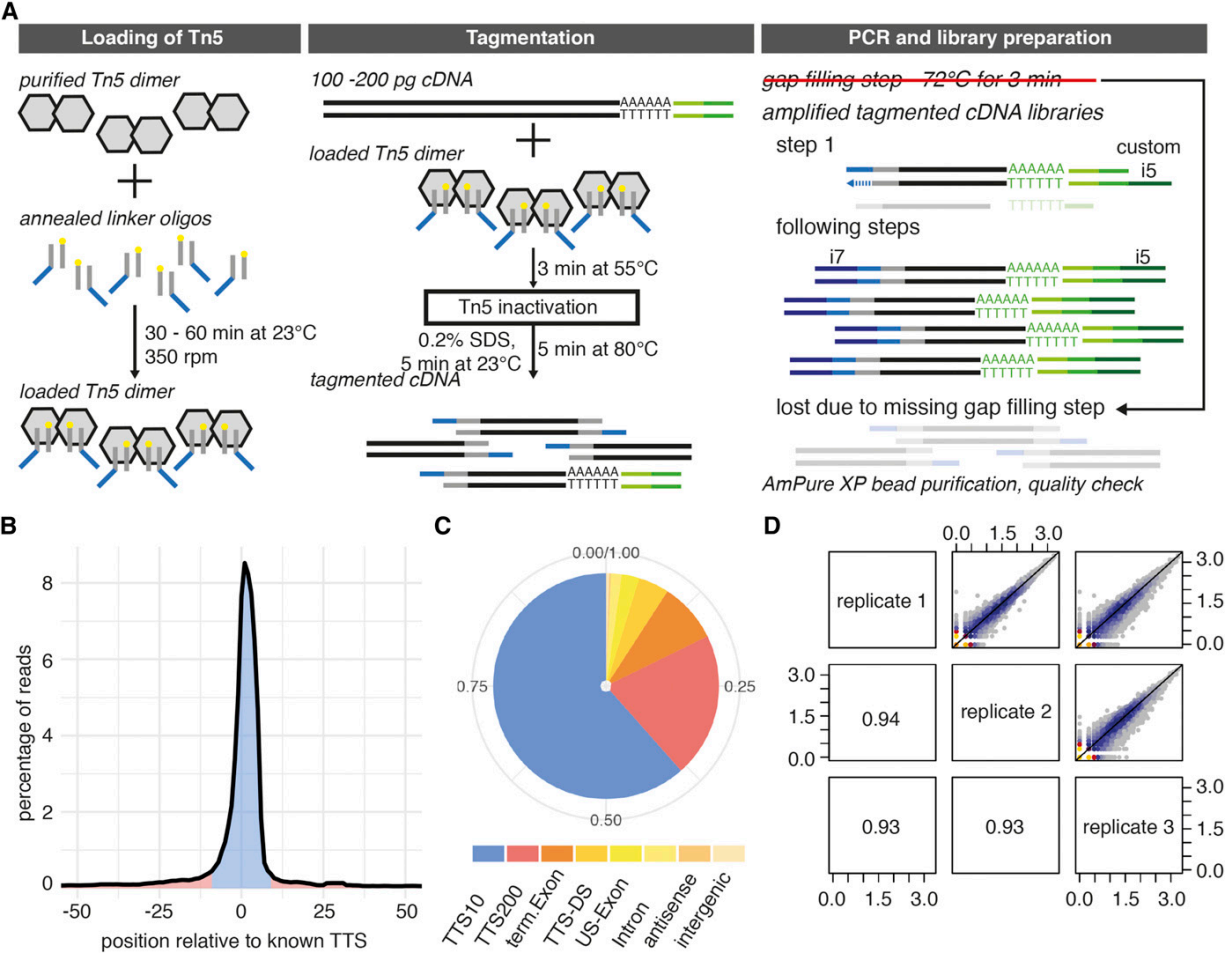
Polyadenylation site mapping in single cells using a customized tagmentation protocol. (A) Workflow of Tn5 loading, cDNA tagmentation, and subsequent NGS library preparation for the mapping of polyadenylation sites in single cells. Tn5 molecules are shown as gray hexamers. The double-stranded part of the linker oligo, the mosaic element, is shown in gray with a yellow circle depicting the phosphorylated 3′ end. The 5′ overhang template for the i7 index adapter primer is shown in blue. cDNA is shown as two parallel black lines with a 3′ poly(A) tail followed by a 6 nt cell barcode (light green) and the template sequence for the customized P5 adapter primer (green, introduced during the priming of the mRNA). In contrast to the duplex index full-length NGS libraries, Tn5 is loaded solely with the linker oligonucleotide for the i7 index adapter primer (blue, i7 index is dark blue). In the subsequent PCR, the initial gap-filling step is omitted so that only the customized i5 adapter primer (dark green) can bind to the cDNA fragments, resulting in amplification from the 3′ end of the cDNA during the first PCR cycle. In the following cycles, PCR enrichment only works for terminal cDNA fragments harboring both adaptors (corresponding to the RNA 3′ end) while the other fragments are lost during library preparation (shown as transparent). (B) Schematic representation of the percentage of reads mapping to the annotated TTS or within the surrounding 50 bp. (C) Distribution of poly(A) reads mapping to different genomic features: TTS, poly(A) signal maps to the TTS of a gene ± 10 or ± 200 bp (TTS10 or TTS200, respectively), poly(A) maps to the terminal exon (term.Exon), poly(A) signal found DS of the TTS (TTS-DS), poly(A) signal is found in the exon before the terminal exon (US-exon), antisense, or intergenic. (D) Pairwise correlation of gene counts between three technical replicates when using in-house-produced Tn5_R27S,E54K,L372P_ and the tagmentation protocol for the mapping of polyadenylation sites in single cells. DS, downstream; NGS, next-generation sequencing; PCR, polymerase chain reaction; SDS, sodium dodecyl sulfate; TTS, transcription termination site; US, upstream.

We modified the established Smart-seq2 protocol (Picelli *et al.* 2014b) by introducing small changes during cDNA processing that allow for specific amplification of only 3′ ends after customized tagmentation with our homemade Tn5. We adapted the oligo dT primer used for mRNA priming and included a 6 nt cell barcode as well as a 4 nt end identifier (“CCAA”). The cell barcode allows for pooling of samples before tagmentation and significantly reduces hands-on time, while the end identifier allows for specific amplification of 3′ ends. mRNA priming and cDNA synthesis were performed as described in Picelli *et al.* (2013) and tagmentation was performed as described above with a few important modifications (see Figure 4A for a schematic workflow): Tn5 was loaded solely with one linker oligonucleotide (Tn5ME-B/Tn5MErev), followed by tagmentation of cDNA using the above-described reaction conditions. In the subsequent PCR, we used the regular i7 adapter index primer but substituted the i5 adapter index primer with a customized one that binds specifically to the end identifier at the 3′ end of the cDNA. To avoid amplification from fragments containing Tn5ME-B adapters at both sites, we skipped the gap-filling step. Without this initial step, the i7 adapter index primer cannot bind the tagmented cDNA, while the customized adapter primer binds its template sequence at the 3′ end of the cDNA. The new, PCR-amplified strand now contains the template sequence for the i7 index adapter so that both primers can bind in the following PCR cycles, resulting in a library suitable for NGS.

We found that 82% of sequencing reads end in poly(A) tails, a much higher fraction than we reported using our previous single-cell polyadenylation site mapping protocol, BATSeq (Velten *et al.* 2015). As described for BATSeq, we then applied further filters to remove short reads, ambiguous alignments, and possible sites of mispriming, yielding 57.3% of reads aligning to high-confidence poly(A) sites (opposed to only 24.3% in BATSeq). On average, 6606 genes were detected per sample (median: 6603, first quartile: 6384, and third quartile: 6833), representing the biological sample well.

As in BATSeq, poly(A) sites identified in single cells mostly corresponded to annotated transcription termination sites (Figure 4, B and C). The correlation of genes detected between samples processed in the same experiment was good, although slightly lower than in the standard full-length cDNA preparation data described above (*r* = 0.93, Figure 4D). Based on these results, we conclude that the modified Smart-seq2 protocol is suitable to successfully process 3′ RNA sequencing libraries with little hands-on time. Notably, the preloaded Nextera ATM enzyme cannot be used in this experimental design as every second cDNA fragment would contain the i5 linker sequence and thereby become lost during PCR amplification. Thus, using the in-house-produced Tn5, NGS library preparation can easily be adjusted and experimental design benefits from customized Tn5 loading strategies.

## Discussion

Here, we present a robust strategy for the purification of Tn5 transposase and a tagmentation-based NGS library preparation protocol. Tagmentation using the His_6_-Sumo3 purified Tn5 results in high-quality NGS libraries with Pearson’s correlation coefficients of 0.99 between samples processed on the same day, and a performance equal to that of the Nextera XT DNA library preparation kit. Importantly, we demonstrate high reproducibility of Tn5 expression and purification in-house and across institutes.

Protein purification via the His_6_-tag is one of the most common strategies in biochemistry as it is robust and easy-to-handle. Consequently, we assume that the required equipment and expertise is available in most biochemistry laboratories, making purification of the Tn5 enzyme straightforward. By contrast, purification of intein-CBD fusion proteins, as described in the published Tn5-intein-CBD purification protocol (Picelli *et al.* 2014a), requires specific chitin beads, which are not commonly found in most biochemistry laboratories. Furthermore, the elution of the protein of interest via on-column cleavage using thiol reagents can take several days and needs to be optimized on a case-to-case basis. The only nonstandard step in our purification protocol is the cleavage of the Sumo3-tag using the SenP2 protease. We will therefore provide the plasmid expressing the His_6_-tagged SenP2 protease upon request. The size exclusion chromatography included in the final step of our purification strategy allows for robust quality control and reproducibility between batches as the dimer peak is exclusively isolated for tagmentation. Any protein aggregates or degradation products are excluded and a pure Tn5 dimer is obtained for every batch of in-house-produced Tn5, ensuring high reproducibility of the enzyme’s activity range in downstream approaches.

In this study, we present two alternative His_6_-Sumo3 purified Tn5 transposases: Tn5_E54K,L372P_ and Tn5_R27S,E54K,L372P_. Both enzymes show remarkable differences in their behavior. Tn5_E54K,L372P_ stably produces fragments with similar size distributions over a wide range of Tn5 concentrations (Tn5-to-input molecule ratio), while Tn5_R27S,E54K,L372P_ carries an additional point mutation (R27S) that is associated with different fragment sizes depending on the Tn5 concentration used. The crystal structure of the Tn5 synaptic complex shows that arginine 27 is involved in the binding of the ME sequence in the linker oligonucleotide that is loaded onto Tn5 (Davies *et al.* 2000). More precisely, arginine 27 forms salt bridges with phosphates in the DNA backbone of the ME sequence. Since the amino acid substitution from arginine to serine neutralizes the positive charge, we hypothesized that the Tn5_R27S,E54K,L372P_ might have a lower affinity to the linker oligonucleotide. To experimentally verify this hypothesis, we performed ITC experiments in which linker oligonucleotide was titrated to either Tn5_E54K,L372P_ or Tn5_R27S,E54K,L372P_. The results of the ITC experiments showed that the equilibrium dissociation constant K_d_ for Tn5_E54K,L372P_ is 0.50 µM, while the K_d_ for Tn5_R27S,E54K,L372P_ is only 12.9 µM (Figure S4 in File S1). The difference in binding affinity might in turn affect the synaptic complex assembly and its functionality. We find this feature intriguing as it makes it possible to customize the fragment length of NGS libraries. The sensitivity of this Tn5 variant for the Tn5-to-input ratio provides further opportunities to develop novel Tn5 applications.

We leveraged the flexible enzyme-loading capacity of our in-house Tn5 to develop a novel method for polyadenylation site mapping in single cells. By loading only one linker oligonucleotide onto the Tn5 enzyme, we ensured that (after tagmentation) all 3′ end fragments of the cDNA contained the correct template sequence for PCR-based library preparation. In contrast, the preloaded Nextera ATM enzyme would introduce both linkers, resulting in a loss of ∼50% of all fragments during PCR amplification. We believe that single-cell approaches with limited input material will benefit from our new method as the comparatively high number of genes detected recapitulates biological samples more accurately.

To date, we have successfully used the in-house-produced Tn5_R27S,E54K,L372P_ transposase and our tagmentation-based library preparation protocol to investigate the differentiation of hematopoietic stem cells based on the transcriptomes of single cells isolated from bone marrow (Velten *et al.* 2017). In addition, our homemade Tn5 variants can be used to generate NGS libraries following the previously published Drop-Seq (Macosko *et al.* 2015) method (see Figure S5A in File S1). Furthermore, NGS libraries can be created from as little as 0.06 pg of genomic DNA by decreasing the Tn5 concentration to 0.6 ng/μl and increasing the number of PCR cycles from 12 to 21, without requiring any further protocol adjustments (Figure S5B in File S1). In conclusion, our Tn5 purification and tagmentation protocol can be used reliably for various experimental set-ups. It presents new options for the field due to its low cost and the high flexibility in experimental design.

## Supplementary Material

Supplemental material is available online at www.g3journal.org/lookup/suppl/doi:10.1534/g3.117.300257/-/DC1.
