# Supplementary material for: Large-Scale Low-Cost NGS Library Preparation Using a Robust Tn5 Purification and Tagmentation Protocol
Source: G3 (Bethesda). 2017 Nov 8;8(1):79–89. doi: 10.1534/g3.117.300257 (PMC5765368; doi:10.1534/g3.117.300257)
Supplement: Supplementary file 1 [file 79FileS1.pdf]

Supplemental material

Supplemental Figures

A)

Tn5$_{E54K,L372P}$

```
 +1
  1 SMITSALHRA ADWAKSVFSS AALGDPRRTA RLVNVAAQLA KYSGKSITIS
 51 SEGSKAMQEG AYRFIRNPNV SAEAIRKAGA MQTVKLAQEF PELLAIEDTT
101 SLSYRHQVAE ELGKLGSIQD KSRGWWVHSV LLLEATTFRT VGLLHQEWWM
151 RPDDPADADE KESGKWLAAA ATSRLRMGSM MSNVIAVCDR EADIHAYLQD
201 KLAHNERFVV RSKHPRKDVE SGLYLYDHLK NQPELGGYQI SIPQKGVVDK
251 RGKRKNRPAR KASLSLRSGR ITLKQGNITL NAVLAEEINP PKGETPLKWL
301 LLTSEPVESL AQALRVIDIY THRWRIEEFH KAWKTGAGAE RQRMEEPDNL
351 ERMVSILSFV AVRLLQLRES FTPPQALRAQ GLLKEAEHVE SQSAETVLTP
401 DECQLLGYLD KGKRKRKEKA GSLQWAYMAI ARLGGFMDSK RTGIASWGAL
451 WEGWEALQSK LDGFLAAKDL MAQGIKI
```

B)

Tn5$_{R27S,E54K,L372P}$

```
 +1
  1 SMITSALHRA ADWAKSVFSS AALGDPRSTA RLVNVAAQLA KYSGKSITIS
 51 SEGSKAMQEG AYRFIRNPNV SAEAIRKAGA MQTVKLAQEF PELLAIEDTT
101 SLSYRHQVAE ELGKLGSIQD KSRGWWVHSV LLLEATTFRT VGLLHQEWWM
151 RPDDPADADE KESGKWLAAA ATSRLRMGSM MSNVIAVCDR EADIHAYLQD
201 KLAHNERFVV RSKHPRKDVE SGLYLYDHLK NQPELGGYQI SIPQKGVVDK
251 RGKRKNRPAR KASLSLRSGR ITLKQGNITL NAVLAEEINP PKGETPLKWL
301 LLTSEPVESL AQALRVIDIY THRWRIEEFH KAWKTGAGAE RQRMEEPDNL
351 ERMVSILSFV AVRLLQLRES FTPPQALRAQ GLLKEAEHVE SQSAETVLTP
401 DECQLLGYLD KGKRKRKEKA GSLQWAYMAI ARLGGFMDSK RTGIASWGAL
451 WEGWEALQSK LDGFLAAKDL MAQGIKI
```

Figure S1: Mass Spectrometry of Tn5 eluates after purification.

Mass spectrometry measurements of **A)** Tn5$_{E54K,L372P}$ and **B)** Tn5$_{R27S,E54K,L372P}$. An excellent protein coverage of 96 % was obtained for both mass spectrometry measurements with all detected amino acids marked in red. The mutated amino acid residues of the hyperactive Tn5 allele are highlighted with a bold black oval (E54K, L372P), while the in this study introduced new mutation, R27S, is highlighted with a bold green oval. The serine residue (black oval) introduced between the Sumo3 protein and Tn5 transposase was not removed during the purification strategy. Tn5 protein coding region starts with the methionine residue on position 2 (arrow, +1).

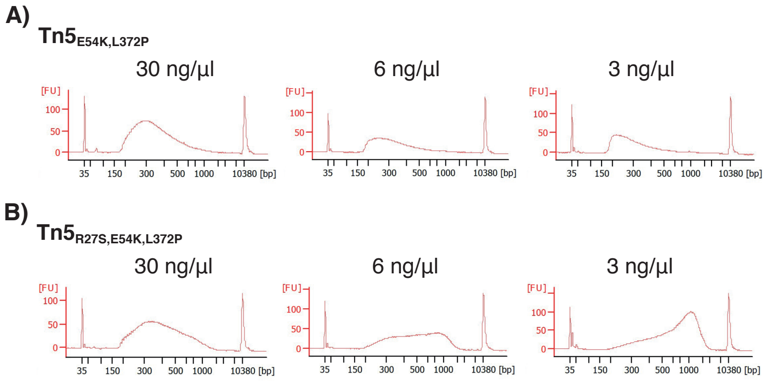

Figure S2: Homemade Tn5 enzymes are active in reagents from Nextera XT DNA library preparation kit.

A) Bioanalyzer traces of NGS libraries processed with different concentrations of in-house produced Tn5$_{E54K,L372P}$ (3 ng/µl – 30 ng/µl). Tagmentation was performed with cDNA at a concentration of approximately 150 pg/µl using all Nextera XT DNA library preparation kit reagents but substituting the ATM enzyme with in-house produced Tn5$_{E54K,L372P}$. A wide range of Tn5$_{E54K,L372P}$ dilutions results in the same average fragment size. **B)** Bioanalyzer traces of NGS libraries processed with different concentrations of in-house produced Tn5$_{R27S,E54K,L372P}$ (3 ng/µl – 30 ng/µl) as described in A).

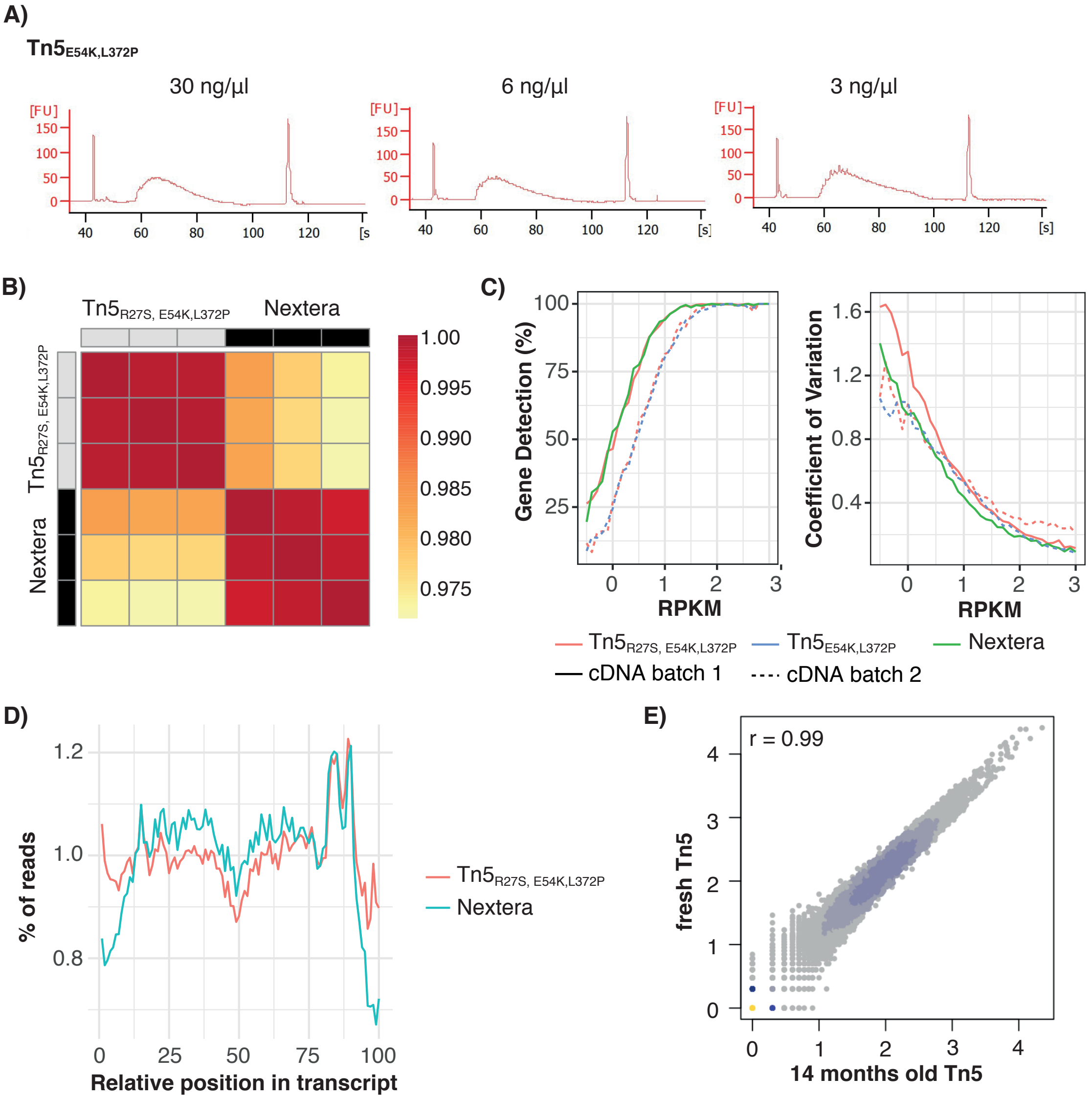

Figure S3: Performance of homemade Tn5 enzymes and the tagmentation protocol.

A) Bioanalyzer traces of NGS libraries processed with different concentrations of in-house produced Tn5$_{E54K,L372P}$ (3 ng/µl – 30 ng/µl). Tagmentation was performed with cDNA at a concentration of approximately 150 pg/µl using the tagmentation protocol developed in this study. **B)** Heat map analysis of gene counts demonstrating high correlation between samples processed with either in-house produced Tn5$_{R27S,E54K,L372P}$ or Nextera XT DNA library preparation kit. Importantly, we detect high correlations between samples processed with either of these enzymes (r > 0.975, see color code on the right side). **C)** Reproducibility of gene expression analysis. The fraction of genes detected in replicate libraries at a given RPKM (left) and the coefficient of variation across replicate libraries at a given RPKM (right) are shown. Genes were binned by RPKM and average values for each bin are shown. **D)** Read coverage over metagene (transcription unit) when using our homemade Tn5 for tagmentation. We generated a metagene of 100 bins in which "0" reflecting the transcriptional start site and "100" the termination site. **E)** Heat scatter showing correlation of read counts between NGS libraries processed with a fresh Tn5 batch or a Tn5 batch stored at -20°C for 14 months. Tagmentation was performed on the same cDNA at a concentration of 150 pg/µl.

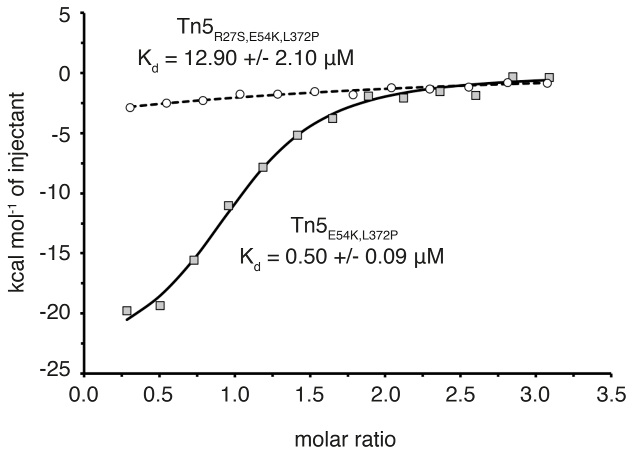

Figure S4: ITC measurement with Tn5 variants and linker oligonucleotides.

Linker oligonucleotide binding to Tn5 measured by isothermal titration calorimetry (ITC) experiments in which 100 µM annealed linker oligonucleotides were titrated to 4 µM $Tn5_{E54K,L372P}$ or $Tn5_{R27S,E54K,L372P}$. The grey squares represent the individual measuring points for $Tn5_{E54K,L372P}$, while circles represent the individual measuring points for $Tn5_{R27S,E54K,L372P}$. Data fitting for $Tn5_{E54K,L372P}$ is shown as full line and for $Tn5_{R27S,E54K,L372P}$ as dotted line.

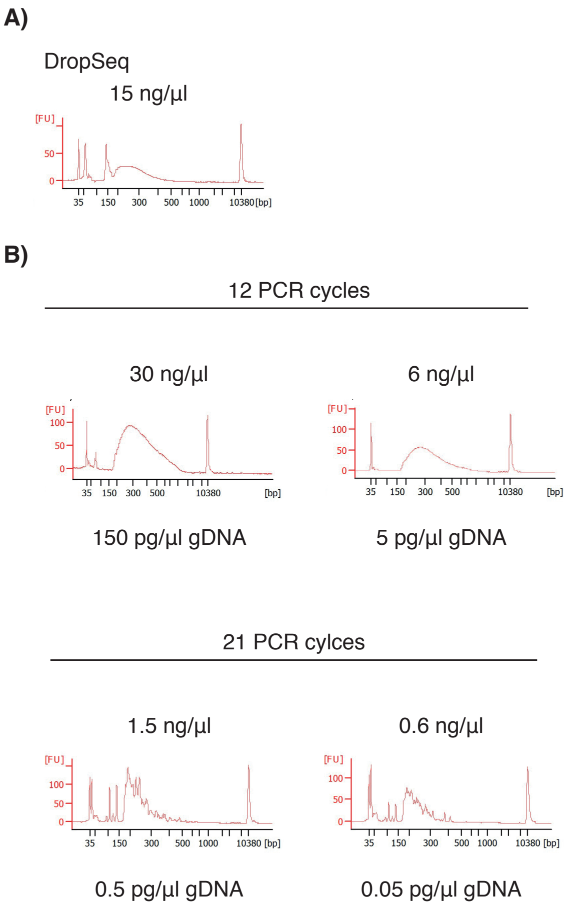

Figure S5: Application of in-house produced Tn5 in various experimental settings.

A) Bioanalyzer traces of Drop-Seq NGS libraries from HEK293T cells processed with homemade Tn5$_{R27S,E54K,L372P}$ at a concentration of 15 ng/µl. **B)** Bioanalyzer traces of NGS libraries processed from genomic DNA at different concentrations ranging from 150 pg/µl to 0.05 pg/µl. Isolated genomic DNA was processed according to the dual-indexed tagmentation protocol presented here (SDS inactivation version). The Tn5$_{R27S,E54K,L372P}$ concentration used is provided above the trace. To account for low input material, the number of PCR cycles was increased from 12 to 21 when processing genomic DNA at concentrations ≤ 0.5 pg/µl.

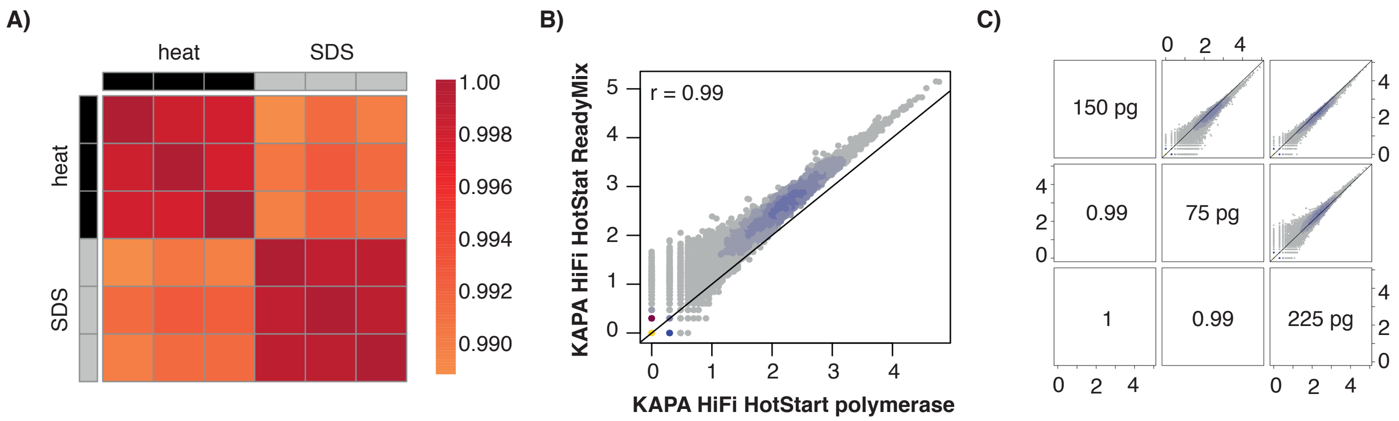

Figure S6: Performance of Tn5 in variations of the tagmentation protocol.

A) Comparison of NGS libraries processed with in-house produced Tn5 and the presented tagmentation protocol but using SDS (final concentration 0.04 %) or heat (80°C for 5 minutes) to inactivate Tn5 after tagmentation. The heat map analysis of gene counts shows very high correlations of r > 0.99 (see color code on the right side), demonstrating that both inactivation methods perform equally well during library preparation. **B)** Heat scatter showing the correlation of read counts between NGS libraries processed with in-house produced Tn5 using either KAPA HiFi HotStart ReadyMix or KAPA HiFi HotStart DNA polymerase for i5/i7 duplex index PCR-based library preparation. **C)** Heat scatter showing the correlation of read counts between NGS libraries processed from cDNA with concentrations ranging from 75 pg/µl – 225 ng/µl (each two samples pooled).

Supplemental methods

Mass Spectrometry

All chemical stock solutions used in this protocol were prepared in 100 mM ammonium bicarbonate buffer pH 8.5 unless stated differently.

Sample preparation

The elution fragments from the SEC runs were analysed via SDS-page. For mass spectrometry analysis, the bands of interest were cut from the SDS-Page with a clean scalpel. Gel pieces were further cut into 1 mm cubes for preparation prior to in-gel digestion. First, the gel pieces were washed with water, then shrunk with acetonitrile for 30 minutes at 56°C prior to reduction using 10 mM DTT. The gel pieces were dehydrated with acetonitrile, followed by an alkylation with 55 mM iodacetamide for 20 minutes in the dark at room temperature. After another dehydration with acetonitrile, the gel pieces were incubated on ice with 1 ng/µL trypsin in 50 mM ammonium bicarbonate, followed by overnight incubation at 37°C. Peptides were extracted from the gel pieces by sonication for 15 minutes. The supernatant was removed and placed in a clean tube. Second extraction was performed as before, with a solution of 50:50 water: acetonitrile, 1 % formic acid (2 x the volume of the gel pieces), and the supernatant was pooled with the first extract. The pooled supernatants were lyophilized via speed vacuum centrifugation. The samples were dissolved in 10 µL of reconstitution buffer (96:4 water: acetonitrile, 0.1% formic acid and analyzed by LC-MS/MS.

LC-MS/MS

Peptides were separated using the nanoAcquity UPLC system (Waters) fitted with a trapping (nanoAcquity Symmetry C18, 5µm, 180 µm x 20 mm) and an analytical column (nanoAcquity BEH C18, 1.7µm, 75µm x 200mm). The outlet of the analytical column was coupled directly to an LTQ Orbitrap Velos (Thermo Fisher Scientific) using the Proxeon nanospray source. Solvent A was water supplemented with 0.1 % formic acid and solvent B was acetonitrile supplemented with 0.1 % formic acid. The samples were loaded with a constant flow of solvent A at 5 µl/min onto the trapping column for 6 minutes. Peptides were eluted via the analytical column at constant flow of 0.3 µl/min. During the elution step, the percentage of solvent B increased in a linear fashion from 3 % to 10 % in 5 minutes, then increased to 40 % in further 10 minutes. The peptides were introduced into the mass spectrometer (Orbitrap Velos Pro, Thermo) via a Pico-Tip Emitter 360 µm OD x 20 µm ID; 10 µm tip (New Objective) and a spray voltage of 2.2 kV was applied. The capillary temperature was set at 300°C. Full scan MS spectra with mass range of 300-1700 m/z were acquired in profile mode in the FT with resolution of 30.000. The filling time was set at maximum of 500 ms with limitation of 106 ions. The most intense ions (up to 15) from the full scan MS were selected for sequencing in the LTQ. Normalized collision energy of 40 % was used and the fragmentation was performed after accumulation of 3 x 104 ions or after filling time of 100 ms for each precursor ion (whichever occurred first). MS/MS data was acquired in centroid mode. Only multiply charged (2+, 3+, 4+) precursor ions were selected for MS/MS. The dynamic exclusion list was restricted to 500 entries with maximum retention period of 30 seconds and relative mass window of 10 ppm. In order to improve the mass accuracy, a lock mass correction using a background ion (m/z 445.12003) was applied.

Data analysis

Acquired data was processed by IsobarQuant (Franken *et al.*, 2015) and Mascot (v2.2.07) and searched against a Uniprot *E. coli* proteome database (UP000000625) containing common

contaminants, reversed sequences and the sequences of the modified proteins. The data was searched with the following modifications: Carbamidomethyl (C) (fixed modification), Acetyl (N-term) and Oxidation (M) (variable modifications). The mass error tolerance for the full scan MS spectra was set to 10 ppm and for the MS/MS spectra to 0.02 Da. A maximum of two missed cleavages was allowed. For protein identification, a minimum of two unique peptides with a peptide length of at least seven amino acids and a false discovery rate below 0.01 were required on the peptide and protein level.

Reference

1. Franken, H., Mathieson, T., Childs, D., Sweetman, G. M., Werner, T., Togel, I., Doce, C., Gade, S., Bantscheff, M., Drewes, G., Reinhard, F. B., Huber, W., Savitski, M. M. (2015) Thermal proteome profiling for unbiased identification of direct and indirect drug targets using multiplexed quantitative mass spectrometry. *Nat Protoc*, **10**, 1567-1593

Expression and purification of $Tn5_{(R27S),E54K,L372P}$

Step-by-step protocol for the expression and purification of the $Tn5_{(R27S),E54K,L372P}$ transposases as well as for the loading of the enzyme and tagmentation-based NGS library preparation.

Reagents and buffers needed

- *E. coli* BL21(DE3) codon + RIL cells (Stratagene)
- LB medium
- Antibiotics: kanamycin (30 mg/ml stock solution; Applichem) and chloramphenicol (10 mg/ml stock solution in 100% ethanol; Applichem)
- Isopropyl-β-D-1-thiogalactopyranoside (IPTG; 1 M stock solution; Peqlab)

- cOmplete protease inhibitor cocktail (Roche)
- 10% polyethylenimine (PEI) pH 7.2 (Sigma)
- 5 ml prepacked cOmplete His-Tag purification column (Roche)
- 12000-14000 MWCO dialysis membrane (Spectra/Por)
- His_6-tagged SenP2 protease (homemade; plasmid and protocols available upon request)
- Superdex200 Increase 10/300 GL column (GE Healthcare)
- Size exclusion chromatography (SEC) buffer: 50 mM Tris pH 7.5, 800 mM NaCl, 0.2 mM EDTA, 2 mM DTT and 10% glycerol
- Running buffer: 20 mM Hepes-NaOH pH 7.2, 800 mM NaCl, 20 mM imidazole, 1 mM EDTA, 2 mM DTT and 10% glycerol
- Elution buffer: 20 mM Hepes-NaOH pH 7.2, 800 mM NaCl, 300 mM imidazole, 1 mM EDTA, 2 mM DTT and 10% glycerol
- Dilution buffer (for storage at -20°C): 100% glycerol and 800 mM NaCl

1. Expression of $Tn5_{(R27S),E54K,L372P}$

A. Add 1 µl of the pETM11-Sumo3-Tn5 construct to 50 µl $CaCl_2$-competent *E. coli* BL21(DE3) codon + RIL cells.

   ** Electro-competent cells can be used as well*

B. Incubate 15 min on ice, 45 sec at 42°C and 5 min on ice

C. Add 1 ml of LB to the transformation mixture and incubate 1 h at 37°C

D. Plate out 100 µl of the transformation mixture on LB-agar supplemented with 30 µg/ml kanamycine and 10 µg/ml chloramphenicol and incubate the plate overnight at 37°C

E. Pick a single colony and inoculate this in 10 ml LB supplemented with 30 µg/ml kanamycine and 10 µg/ml chloramphenicol in a 50 ml shaking flask

   <u>Note:</u> *In case of problems with the expression of the Tn5 protein: try to inoculate the transformed E. coli cells directly in 10 ml LB supplemented with 1% glucose, 30 µg/ml kanamycine, and 10 µg/ml chloramphenicol in a 50 ml shaking flask*

F. Shake the preculture overnight at 37°C and 200 rpm

G. Add 10 ml preculture to 1 liter LB supplemented with 30 µg/ml kanamycine and 10 µg/ml chloramphenicol in a 5 l shaking flask

H. Grow the culture at 37°C and 200 rpm until OD_{600} ~ 0,5. Then reduce the temperature to 18°C and allow the culture to cool down for 30 min.

I. Induce the production of recombinant Tn5 by adding 0.2 mM IPTG to the culture. Allow the cultures to continue growing overnight at 18°C.

J. Harvest the cells by centrifugation (4600 x *g*, 30 min, 4°C) and store the pellet at -20°C if you don´t continue with the purification immediately.

2. Purification of $Tn5_{(R27S),E54K,L372P}$

A. Resuspend the cell pellet from 1 liter culture in 50 ml running buffer supplemented with cOmplete protease inhibitors.

B. Lyse the cells via sonication. We use a Branson sonicator with a 10 mm tip and 4-5 cycles of 30 sec with intermittent cooling with a 50% duty cycle at output 5-6.

** Sonification can be replaced by using the M-110L Microfluidizer (Microfluidics)*

C. Centrifuge the lysate (30000 x *g*, 30 min, 4°C).

D. Add 6 ml of 10% PEI pH 7.2 dropwise to the cleared lysate while constantly stirring the solution.

** PEI removes nucleic acids from the lysate and is required to avoid contamination of E. coli DNA (bound to the Tn5 protein).*

E. Centrifuge the lysate (20000 x *g*, 30 min, 4°C) to remove the PEI-precipitated fraction.

F. Equilibrate a 5 ml prepacked cOmplete His-Tag purification column with running buffer at a flow rate of 1 ml/min.

G. Load the cleared lysate onto the equilibrated cOmplete His-Tag purification column at a flow rate of 0.7 ml/min.

** It is important to use the cOmplete His-Tag purification column as it is resistant to DTT and EDTA. Nickel gets stripped off the beads when using a Ni-NTA column.*

H. After loading the sample, wash the cOmplete His-Tag purification column with running buffer until the UV_280nm signal returns to baseline at a flow rate of 1 ml/min.

I. Elute the His_6-Sumo3-tagged Tn5 from the cOmplete His-Tag purification column in a one-step elution using elution buffer at a flow rate of 1 ml/min. Collect the eluate in 2 ml fractions.

J. Analyse the elution fractions via SDS-PAGE and pool the fractions containing His_6-Sumo3-tagged Tn5.

K. To remove the His_6-Sumo3 tag, add His_6-tagged SenP2 to the pooled elution fractions in a 1:100 ratio and dialyse the mixture overnight to running buffer at 4°C.

L. Load the dialysate on a 5 ml cOmplete His-Tag purification column equilibrated with running buffer at a flow rate of 0.7 ml/min. The uncleaved His_6-Sumo3-Tn5 fusion protein, the cleaved off His_6-Sumo3 tag and the His_6-SenP2 will bind to the column, while the untagged Tn5 can be collected in the flow through of the column.

M. Analyse the samples via SDS-PAGE and pool the Tn5-containing fractions.

N. Concentrate the sample to an appropriate volume for the subsequent size exclusion chromatography (SEC). In our hands the SEC worked best by loading maximum 3 mg of Tn5 per run in a volume of maximum 500 µl.

O. Equilibrate the Superdex200 (Increase) 10/300 GL column with SEC buffer.

   ** a Superdex75 (Increase) 10/300 GL column can be used as an alternative in case there is no Superdex200 (Increase) 10/300 GL column available.*

P. Inject 500 µl sample containing ~ 3 mg untagged Tn5. Use a flow rate of 0.5 ml/min and a fraction size of 500 µl for the SEC runs.

Q. The Tn5 elutes in 2 peaks (figure 1C and 1D): the first peak corresponds to the void volume of the column and contains most likely Tn5 aggregates. The second peak appears at approximately 12.9 ml for $Tn5_{R27S,E54K,L372P}$ and at 13.2 ml for $Tn5_{E54K,L3729P}$, which corresponds to the molecular weight of a Tn5 dimer (molecular weight of a Tn5 monomer: 53 kDa).

R. Analyse the elution fractions via SDS-PAGE and pool the Tn5-containing fractions of the second elution peak of consecutive SEC runs.

S. Determine the concentration of the final sample. In case you plan on storing the aliquots at -20°C, dilute the final sample 1:1 with a buffer containing 100% glycerol and 800 mM NaCl. The final yield of Tn5 dimer varied between 1-5 mg per liter culture. The working stock concentrations were in the range of 0.2 mg/ml to 0.4 mg/ml (in the manuscript referred to as “Tn5 stock sample, undiluted”).

T. Aliquot the samples and store them in 25 mM Tris pH 7.5, 800 mM NaCl, 0.1 mM EDTA, 1 mM DTT and 50% glycerol at -20°C. In case the samples need to be shipped, they can be flash-frozen with liquid nitrogen in the SEC buffer as well and stored at -80°C.

All chromatography steps were performed at 4°C on an Åkta Purifier 10 or on an Åkta Pure 25 chromatography system (both from GE Healthcare). To preserve the activity of the protein, it´s important to keep the sample cold throughout the entire purification process.

Tn5$_{(R27S),E54K,L372P}$ loading and tagmentation-based NGS library preparation

This protocol describes the workflow of the Tn5 loading and tagmentation-based library preparation for dual indexing i5/i7 NGS. Details specific to the 3'RNA-Seq protocol are provided as additional information and marked in green.

Reagents and buffers needed

- Annealing buffer: 50 mM NaCl, 40 mM Tris-HCl pH 8.0
- 4x Tagmentation buffer: 40 mM Tris-HCl pH 7.5, 40 mM $MgCl_2$
- 100 % DMF (Sigma Aldrich)
- 0.2 % SDS
- KAPA HiFi HotStart ReadyMix or KAPA HiFi HotStart polymerase
- 100 % DMSO
- AMPure XP beads (Beckman Coulter)
- 80 % freshly prepared ethanol
- Illumina i5 and i7 adapter index primers
- Custom PE1.3'RNA-Seq i5 adapter index primer
- Nuclease-free water
- Qubit HS dsDNA kit (Thermo Fisher Scientific)
- High Sensitivity DNA kit (Agilent)

All oligonucleotides were ordered at HPLC grade from Sigma Aldrich, Germany.

1. Annealing of the linker oligonucleotides Tn5ME-A/Tn5MErev and Tn5ME-B/Tn5MErev

A. Resuspend lyophilized oligonucleotides in annealing buffer (50 mM NaCl, 40 mM Tris-HCl pH 8.0) to a stock concentration of 100 μM and mix one volume of Tn5ME-A or Tn5ME-B with one volume of Tn5MErev (working stock, 50 μM). Distribute the mix in 10-20 μl aliquots for storage at -20C.

Tn5ME-A 5′-TCGTCGGCAGCGTC<u>AGATGTGTATAAGAGACAG</u>-3′

Tn5ME-B 5′-GTCTCGTGGGCTCGG<u>AGATGTGTATAAGAGACAG</u>-3′

Tn5MErev 5′-[phos]<u>CTGTCTCTTATACACATCT</u>-3′

**ME sequence that is bound by the Tn5 is underlined*

B. Run the following PCR program in a thermocycler for the annealing of the oligonucleotides:

95°C 5 min

slowly cool down to 65°C (0.1°C/sec)

65°C 5 min

slowly cool down to 4°C (0.1°C/sec)

C. Store the annealed linker oligonucleotides at -20°C.

2. Loading of the Tn5$_{(R27S),E54K,L372P}$ with Tn5ME-A/Tn5MErev and Tn5ME-B/Tn5MErev

A. Thaw the annealed linkers on ice. Add 0.5 μl of each annealed linker at a concentration of 35 μM to 10 μl of the Tn5 stock (0.2 mg/ml - 0.4 mg/ml). Mix well.

*** Use 1 μl of the 35 μM Tn5ME-B/Tn5MErev linker oligonucleotide but not the Tn5ME-*

A/Tn5MErev when loading the Tn5 for the 3’ RNA Seq protocol

B. Incubate linker-Tn5 mix at 23°C under constant shaking at 350 rpm in a thermomixer for 30-60 min.

**Make sure to keep the temperature constant at 23°C*

**Do not exceed 60 minutes of loading as the Tn5 enzyme will gradually loose activity*

**Proceed immediately with the tagmentation reaction or supplement the loaded Tn5 with glycerol to a final concentration of 50 % and store at -20°C for several days.*

C. Tn5 dilutions can be prepared with nuclease-free water. A final concentration in the range of 20-40 ng/μl is suitable for the tagmentation of cDNA to fragment sizes of 350 bp on average.

** An initial dilution series of every new batch of Tn5 might be beneficial to check the Tn5 activity range*

3. Tagmentation-based library preparation

A. Dilute the cDNA in nuclease-free water to a concentration of 100 pg/μl – 200 pg/μl

** see figure S6c for Pearson correlations of libraries processed with different cDNA concentrations.*

B. Mix one volume of the 4x tagmentation buffer with one volume of 100 % DMF, referred to as tagmentation mix

** The tagmentation mix should be prepared fresh. As DMF is unstable in solution, we find decreased Tn5 activity in tagmentation mix prepared 30 minutes prior to experimentation*

C. Assemble the tagmentation reaction and mix well:

2.50 µl tagmentation mix

1.25 µl 100 pg/µl – 200 pg/µl cDNA

1.25 µl Tn5 at desired concentration

5.00 µl in total

D. Perform tagmentation reaction with the desired inactivation method, SDS or heat (see figure S6a).

** make sure that the thermocycler is pre-heated to 55°C*

** SDS inactivation results in higher yields after PCR enrichment*

** heat inactivation ensures same tagmentation time for all samples in large scale experiments and reduces hands-on time.*

<table>
<tr><th>SDS inactivation</th><th>heat inactivation</th></tr>
<tr><td colspan="2">55°C for 3 minutes in a pre-heated thermocycler</td></tr>
<tr><td>cooling down to 10°C</td><td>80°C for 5 min in a thermocycler</td></tr>
<tr><td>add 1.25 µl 0.2 % SDS</td><td rowspan="2">cooling down to 10°C</td></tr>
<tr><td>incubate at room temperature for 5 min</td></tr>
<tr><td colspan="2">PCR enrichment (KAPA HiFi polymerase or KAPA HiFi HotStart ReadyMix)</td></tr>
</table>

E. Add 10 µl of the PCR mastermix to each sample and run the following PCR program

** KAPA HiFi HotStart ReadyMix or KAPA HiFi HotStart polymerase can be used interchangeable (figure S6b)*

** for multiplexing: Add a unique pair of i5 and i7 adapter index primers separately to each sample.*

*** Use KAPA HiFi HotStart polymerase mastermix when processing 3'RNA Seq libraries and exchange 0.75 µl DMSO with 1.00 µl 0.5 M TMAC. Add 10.25 µl PCR mastermix to the samples. Also, use the customized i5 adapter index primer (PE1.3'RNA-Seq) instead of the Illumina i5 adapter index primer.*

**KAPA HiFi HotStart ReadyMix**	**KAPA HiFi HotStart polymerase**
	3.00 µl 2x KAPA buffer
6.75 µl 2x KAPA ReadyMix	0.75 µl 100 % DMSO
0.75 µl 100 % DMSO	1.25 µl 10 µM i5 adapter index primer
1.25 µl 10 µM i5 adapter index primer	1.25 µl 10 µM i7 adapter index primer
1.25 µl 10 µM i7 adapter index primer	0.45 µl 10 mM dNTPs
**10.00 µl in total**	0.30 µl KAPA HiFi HotStart polymerase
	3.00 µl nuclease-free water
	**10.00 µl in total**

** the gap filling step at 72°C for 3 min is essential to filling the 5' overhangs of the single stranded linker oligonucleotides to allow for the binding of adapter primers and the amplification of the tagmented cDNA library. Make sure to skip the gap-filling step in the 3'RNA Seq protocol.*

i5/i7 libraries	3’RNA-Seq
72°C 3 min	
95°C 30 sec	95°C 3 min
98°C 20 sec	98°C 20 sec
58°C 15 sec 12 cycles	62°C 15 sec 12 cycles
72°C 30 sec	72°C 30 sec
72°C 3 min	72°C 3 min
10°C hold	10°C hold

** Increase number of cycles if starting from ultra low input material. We increased the number of cycles to 21 if processing input material ≤ 0.5 pg/µl, while 12 cycles suitable for processing libraries from 5 pg/µl input material.*

F. Remove residual dNTPs, primers, and the polymerase by adding one volume of AMPure XP beads to the sample (1:1 v/v) and follow the manufacturers instructions. Elute with 10 µl nuclease-free water.

G. Determine the concentration of the tagmented cDNA libraries using Qubit HS dsDNA and check the quality of the libraries on the Agilent 2100 Bioanalyzer using the Agilent High Sensitivity DNA Kit.

Table S1: Primers used in this study

PRIMER	SEQUENCE	APPLICATION
Tn5-fw (*Bam*HI)	5'-GATCGGATCCATGATTACCAGTGCACTGCATCG-3'	subcloning of Tn5 allele
Tn5-rev (*Hin*dIII)	5'-GATCAAGCTTTTAGATTTTAATGCCCTGCGCC-3'	
oligo dT	5'-AAGCAGTGGTATCAACGCAGAGTAC**CCAA***CACTGT* NNNNNNNVTTTTTTTTTTTTTTTTTTTTVN-3'	polyA priming (based on Picelli et al. 2014b) end identifier: bold barcode: italic
TSO	5'-AAGCAGTGGTATCAACGCAGAGTACATrGrG+G-3'	Template switching (Picelli et al. 2014b) ordered from IDT
Tn5ME-A	5′-TCGTCGGCAGCGTCAGATGTGTATAAGAGACAG-3′	Linker oligonucleotides for tagmentation
Tn5ME-B	5′-GTCTCGTGGGCTCGGAGATGTGTATAAGAGACAG-3′	
Tn5MErev	5′-[phos]CTGTCTCTTATACACATCT-3′	
PE1.Smart-seq2	5'-AATGATACGGCGACCACCGAGATCTACACAAGCAG TGGTATCAACGCA GAGTACCCAA-3'	Amplification of 3'RNA-Seq libraries
polyA-seq	5'-AAGCAGTGGTATCAACGCAGAGTACCCAA-3'	3'RNA-Seq sequencing primer
